# Accurate deployment of self-expanding transcatheter aortic valve implantation for permanent pacemaker reduction

**DOI:** 10.1016/j.xjse.2024.100013

**Published:** 2024-07-14

**Authors:** Joe Aoun, Sachin S. Goel, Michael J. Reardon

**Affiliations:** a Department of Cardiology, Houston Methodist Hospital, Houston, Tex; b Department of Cardiovascular Surgery, Houston Methodist Hospital, Houston, Tex

**Figure:**
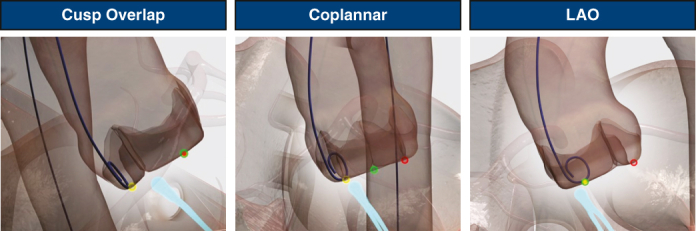
Cusp overlap technique.

**Central Message**

Pacemaker rate is related to TAVI technique. The rate can be reduced using the cusp overlap technique.

The cusp overlap implant technique (Figure 1 and Video 1) stands as a significant advancement in transcatheter aortic valve implantation (TAVI) with self-expanding valves, aimed at reducing the need for permanent pacemaker implantation postprocedure.^1^, ^2^, ^3^ The Video provides an overview of the cusp overlap technique, including steps to ensure proper commissural alignment during TAVI using the Evolut FX platform (Medtronic).

**Figure 1:**
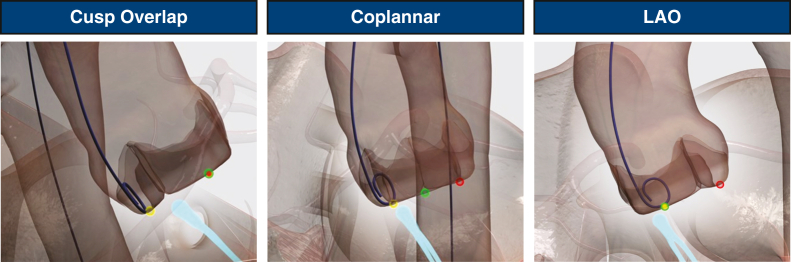
TAVI angles. *LAO*, Left anterior oblique.

Preceding the intervention, careful preprocedural planning is essential, involving the determination of the cusp overlap angle through computed tomography, typically viewed from a right anterior oblique/caudal perspective. This technique involves isolating the noncoronary cusp and overlapping the left and right coronary cusps to precisely position the valve during deployment, ensuring a consistent depth of 3 mm on the noncoronary cusp side.

As the delivery system is advanced, it is crucial to keep the flush port positioned at 3 o'clock. This ensures that the hat marker aligns with the outer curve of the descending aorta when viewed from a left anterior oblique perspective, thus aiding in commissural alignment during valve deployment. Upon reaching the annulus, the hat marker ideally should be in a center front position in a cusp overlap view (and at the outer curve in a coplanar view). During the deployment phase, a careful and slow approach is recommended, often conducted under controlled pacing (100-140 bpm).

Any necessary adjustments to the valve position are made with careful consideration, either by advancing the system forward or pushing the Safari wire forward if the valve is too deep. With regard to the Evolut FX platform, it is crucial to ensure that the dot is positioned at the base of the noncoronary cusp to achieve an implantation depth of 3 mm. Postdeployment, meticulous attention is required for safe removal of the nose cone, mitigating potential complications such as valve migration.

Commissural alignment is verified by ensuring that there are 2 dots at the noncoronary cusp position and 1 dot at the left cusp position in the cusp overlap view. In conclusion, the proper implant depth achieved through the cusp overlap technique is essential for reducing the risk of permanent pacemaker dependency in modern TAVI procedures involving self-expanding valves.

## Conflict of Interest Statement

S.S.G. is a consultant for Medtronic, JC Medical, and WL Gore & Associates and is also on the Speakers Bureau for Abbott Structural Heart. M.J.R. discloses consultancy roles with Medtronic, Boston Scientific, Abbott, and WL Gore & Associates. The other author reported no conflicts of interest.

The *Journal* policy requires editors and reviewers to disclose conflicts of interest and to decline handling or reviewing manuscripts for which they may have a conflict of interest. The editors and reviewers of this article have no conflicts of interest.

## Supplementary Data

Video 1 Accurate deployment of self-expanding TAVI for permanent pacemaker reduction. Video available at: https://www.jtcvs.org/article/S2950-6050(24)00013-5/fulltext.

## References

[1] Wienemann H., Maier O., Beyer M. (2023). Cusp overlap versus standard three-cusp technique for self-expanding Evolut transcatheter aortic valves. EuroIntervention.

[2] Tang G.H.L., Zaid S., Michev I. (2018). “Cusp-overlap” view simplifies fluoroscopy-guided implantation of self-expanding valve in transcatheter aortic valve replacement. JACC Cardiovasc Interv.

[3] Jilaihawi H., Zhao Z., Du R. (2019). Minimizing permanent pacemaker following repositionable self-expanding transcatheter aortic valve replacement. JACC Cardiovasc Interv.

