# Supplementary figures and images for: Accurate deployment of self-expanding transcatheter aortic valve implantation for permanent pacemaker reduction
Source: JTCVS Struct Endovasc. 2024 Jul 14;1-2:100013. doi: 10.1016/j.xjse.2024.100013 (PMC13244767; doi:10.1016/j.xjse.2024.100013)

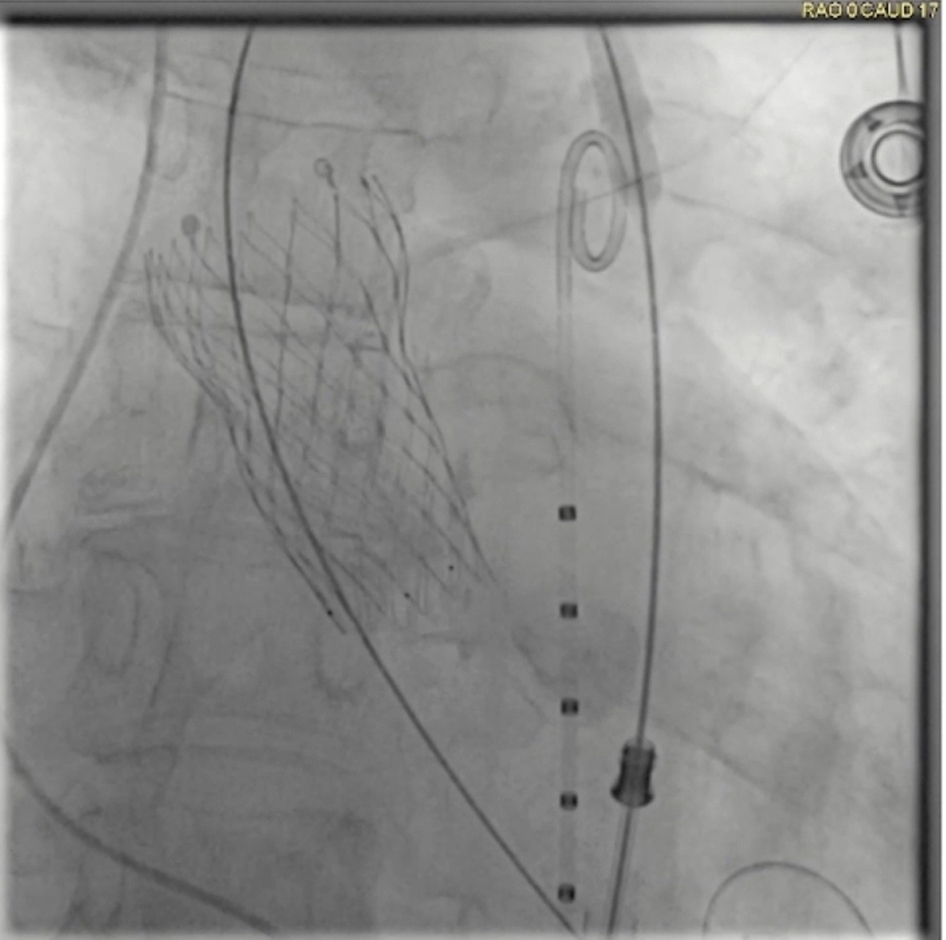

Supplement: Video 1 — Accurate deployment of self-expanding TAVI for permanent pacemaker reduction. Video available at: https://www.jtcvs.org/article/S2950-6050(24)00013-5/fulltext. [file fx2.jpg]
